# Exploring spatial uncertainty in HIV risk locations: individual characteristics and geographic reporting accuracy

**DOI:** 10.1186/s12889-026-27988-9

**Published:** 2026-06-17

**Authors:** Sofia Kaloper, Alan T. Murray, Sean Reid, Pamina Gorbach, Susan Cassels

**Affiliations:** 1 Department of Geography, University of California, Santa Barbara, Santa Barbara, CA USA; 2 Department of Epidemiology, University of California, Los Angeles, Los Angeles, CA USA

**Keywords:** Spatial Uncertainty, HIV Risk Hotspots, Activity Spaces, Location-based Survey Methods, Geographic Information Systems (GIS)

## Abstract

This paper investigates the determinants of spatial uncertainty associated with respondent reporting of potential HIV risk activity, lifestyle, and healthcare locations in Los Angeles. We examine which geographic and socio-demographic variables are associated with the accuracy of location reporting. Significant variables associated with activity space reporting accuracy related to HIV risk and HIV prevention are identified. The study is based on gay, bisexual, and other sexual minority men (SMM) enrolled in the UCLA mSTUDY, collecting demographic characteristics and locations of recent activities. An exploratory spatial data analysis is carried out. Statistical modeling with logistic regression was used to identify sources of uncertainty and their influence on reporting accuracy. This paper concludes that demographic characteristics, activity types, and geographic location are significant predictors of reported geographic accuracy. The research also found regional variation in accuracy, with locations within regions reported with higher accuracy than those outside them. Future work aims to refine statistical models of location accuracy and establish confidence intervals to mitigate uncertainty, which is needed to improve the effectiveness of interventions aiming to reduce HIV incidence disparities.

## Introduction

Even with global and national declines in HIV incidence rates, gay, bisexual, and other sexual minority men (SMM) disproportionately bear the burden of new infections, especially among young Black and Hispanic men [1]. Intervention strategies must therefore be further refined to overcome racial and ethnic disparities in HIV infection rates. Previous work has demonstrated that racial and ethnic minority SMM tend to experience greater fragmentation in activity spaces as sexual encounters, social interactions, and residential life often occur in very different places [2–4]. Hotspots are locations with a higher value of a phenomenon relative to its surroundings, given a random distribution of events [5]. In the context of HIV prevention, hotspots are geographic locations with an elevated HIV incidence relative to surrounding areas. Current methods only consider hotspots of HIV diagnoses, not risk hotspots or areas with elevated infection risk. Interventions guided by HIV incidence hotspots, therefore, fail to account for the fragmented activity spaces of minority SMM populations, thereby exacerbating HIV incidence disparities.

While health disparity research has acknowledged neighborhood effects on health, the fragmented neighborhoods of SMM have been generally overlooked. Activity spaces [6–8] are defined as a set of locations where an individual routinely engages in social interaction of some sort, creating exposure and influence. Life takes place not just within the home, but in the places, networks, environments, and social spaces individuals visit. Activity spaces play a vital role for SMM as social and sexual interactions often occur outside their residential neighborhood [4, 9–11]. By examining where different risk activities occur, prevention can better target HIV incidence among SMM [12, 13].

While place effects have long been acknowledged and studied [14, 15], spatial stigma has emerged as a newly conceptualized mechanism through which place affects health. Spatial stigma refers to the processes by which an individual is associated with and reduced to the perception of the neighborhood in which they reside [16, 17]. It has been acknowledged as a potential mechanism by which the place effects can “get under the skin” through three primary pathways: access to material resources, processes of stress and coping, and processes related to identity formation and management [17].

HIV risk hotspots reflect the geographic locations and network interactions of substance use and sexual activity. However, precisely identifying and capturing the locations of these stigmatized activities is challenging. Most approaches for identifying hotspots are inherently uncertain in various ways. Spatial uncertainty is the lack of or error in geographic position, and can be introduced as a result of poor understanding [18] or geocoding (Delmelle et al. 2022). Errors introduced through misunderstanding may result from a limited grasp of the relevant issues, incorrect interpretation of the issues, or implementation of an inappropriate method or model [18].

Of course, another source of spatial uncertainty is incorrect or imprecise reporting of locations. This spatial uncertainty can be introduced both intentionally and unintentionally. Intentional response errors generally stem from issues of privacy. Individuals may not feel comfortable disclosing the locations of stigmatized activities, such as substance use locations or the locations of their sexual partners. Unintentional response errors can stem from a failure to remember the exact location, errors in the location recording process (such as technical difficulties or accidentally selecting the wrong location), and from errors in the geocoding of addresses.

An additional source of spatial uncertainty stems from the intentional introduction of uncertainty by researchers. In order to share data outside of a host institution without violating respondent confidentiality and still maintaining locational geographic integrity, geomasking techniques allow researchers to introduce a small degree of uncertainty without overtly influencing the resulting analyses performed on masked datasets [19–22]. These masking techniques allow for statistical methods to account for the locational uncertainty of the geocoded data so that analysis still yields robust results. These methods also help maintain individual privacy by preventing individuals from being identified by their exact geo-spatial locations.

Reducing HIV incidence disparities relies on the identification of geographic risk hotspots to allow for targeted interventions through innovative place-based interventions [3], such as mobile HIV testing vans, collaboration with community spaces, or satellite HIV care clinics in neighborhoods where no HIV care exists. HIV hotspots provide clear geographic locations for intervention strategies, and the network connectivity of hotspots can allow for the prioritization of one hotspot over another. However, in order for hotspots to be useful in guided interventions, spatial uncertainty must be accounted for. A key principle in hotspots is that high values of the phenomenon are spatially clustered together, producing areas having a higher rate of the phenomenon compared to its neighbors [5]. Because the spatial location of the phenomena is fundamental to hotspot analysis, spatial uncertainty is critical to account for. However, the analysis methods upon which hotspots are identified rely on the assumption that the amount of spatial uncertainty within the data is 1) fairly small and uniform, and 2) distributed throughout the dataset independently of the outcome variable.

Although methods exist to account for the amount of geo-spatial uncertainty in analysis [23], these methods must know the amount of uncertainty in advance. In regards to respondent reported locations, the true degree of uncertainty is always unknown, as is the actual distribution of the uncertainty within the dataset. This paper seeks to address whether HIV risk and prevention locations are reported with evenly distributed spatial uncertainty across different demographic and contextual characteristics.

The aim of this paper is to examine social and contextual characteristics associated with reporting uncertainty of activities in Los Angeles. In order to reduce HIV disparities and incidence, interventions should be targeted to HIV risk hotspots. However, spatial uncertainty in the data used to identify hotspots can result in inaccurate geographic identification. Without accurate hotspots, interventions targeting risk areas are likely to fall short of their goals and contribute to growing HIV incidence disparities. Location-based interventions need to be accurate to be effective, and while some level of spatial uncertainty is inevitable, supporting methods must address the impact of uncertainty. This paper seeks to assess if and how geographic, socio-demographic, and activity-specific characteristics are associated with location reporting accuracy.

## Methods

### Data

The analysis focuses on HIV risk associated with SMM living in the greater Los Angeles region. The data were collected through a survey of individuals enrolled in the UCLA mSTUDY [24, 25], a NIDA-funded cohort of SMM living with and without HIV that enrolled participants from 2014 to 2024 and collected detailed behavioral and biological data from them at semi-annual visits. The data gathered from the participants included demographic, life event, mobility, housing, and geospatial information for each respondent. The demographic information collected provided details on the respondent’s race, education, employment, and familial characteristics. The life event questions collected information about the respondent’s sexual behavior, demographic characteristics, and lifestyle behaviors (including questions related to the outness of a respondent’s sexuality). The mobility and housing questions collected information on housing instability, household composition, and transportation modes of respondents. The geospatial information contained locations for the participant’s current home, home 12 months ago, home 5 years ago, medical care, pharmacy, location of the most recent HIV test, location of the most recent STD test, primary and secondary social locations, three most recent locations of substance use and the location of most recent sexual activity with the partner’s three most recent sexual partners. The geospatial information also collected details on respondents’ substance use behaviors. The data used in this study consisted of an analytical subsample of the original mSTUDY dataset. Participants who reported locations outside of Los Angeles were excluded (13 respondents), resulting in a final sample of 2,112 locations reported by 206 unique participants.

The geospatial information was collected from a three-level survey design based on the nature of the locations participants were asked to identify. When identifying a location, participants would first identify a general area from a map of the large, generalized regions within Los Angeles County. The participants would then identify the neighborhood in which the activity was located from a map zoomed into that region indicating possible neighborhoods. Finally, in the third level, participants were presented with an embedded Google Maps interface zoomed into the center of the identified neighborhood [24]. Participants were able to mark the location on the embedded map by either dragging and dropping a pin or typing the name or address of the location in a Google Maps search bar. After marking the location, participants answer a question providing information about the accuracy in which they marked the location. Participants could select from seven prespecified options: “I marked the exact location on the map”, “I moved the pin within one block of this location”, “I moved the pin further than one block from this location”, “I do not feel comfortable marking this location for privacy reasons”, “I can’t remember the exact location, but marked the general areas”, “I can’t remember the exact location” and “I’m having trouble using the map to mark the exact location”. Participants were also presented with an eighth option to describe how they marked the location “Other (Please specify below)”, which would allow the participant to provide their own written description. Participants would assign each location a nickname, then, given the option later in the survey, were asked if the activity was located at any of the previously marked locations, or if the activity occurred at a new location.

Each location was stored as a point and contained attribute information designating a unique study ID of the participant (PID). This allowed the participant’s identity to remain anonymous, but provides links to the general activity type of the location (i.e., home, social, substance, sex, etc.) and a term to account for the single activity type category (i.e., sexual partner 1 location, sexual partner 2 location, primary social location, home 12 months ago, etc.). The point also contained the name (and ID) of the region selected by the participant in the first map level, the name (and ID) of the neighborhood selected in the second map level, and the accuracy reported by the participant in marking the location.

### Analytic approach

**Fig. 1:**
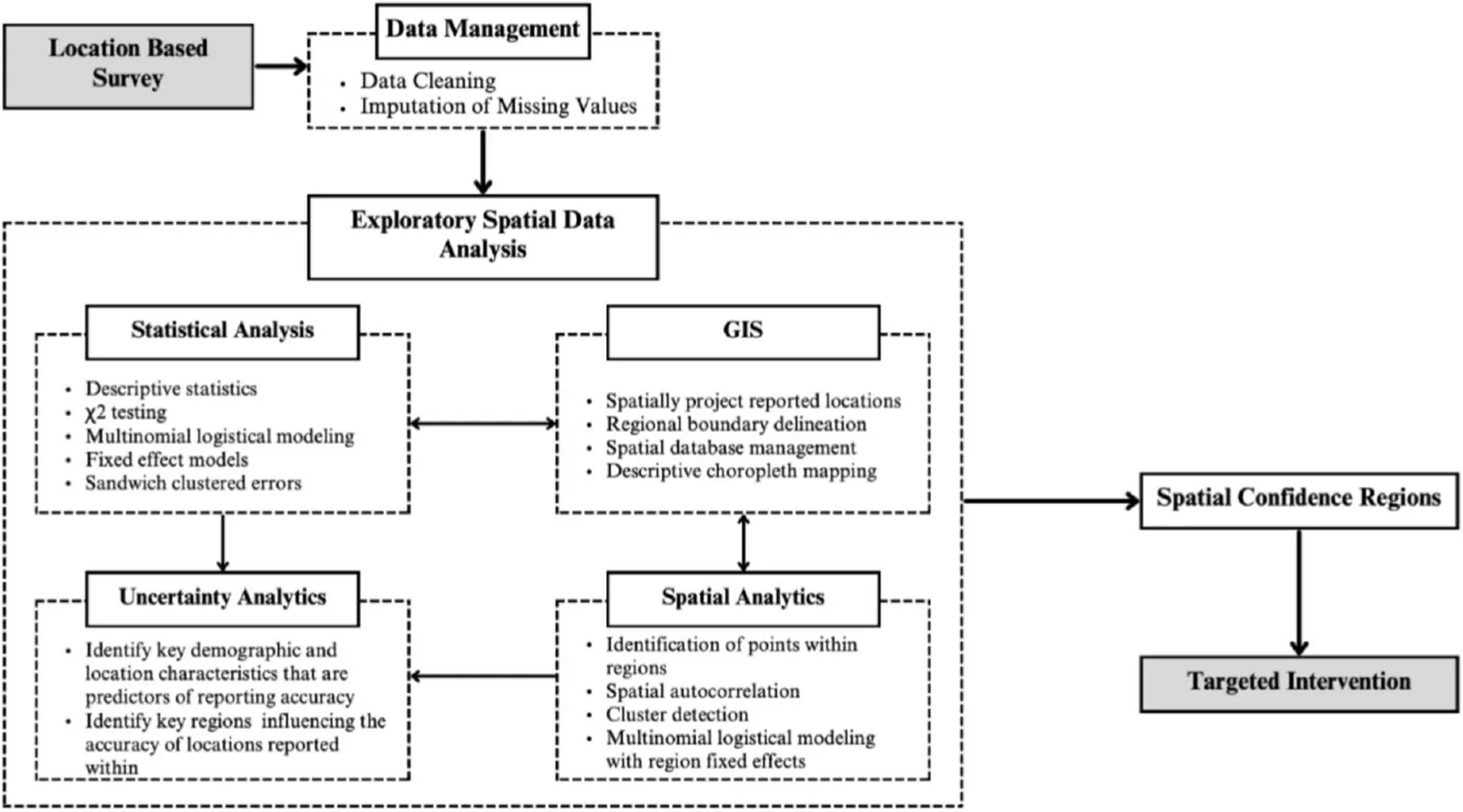
Spatial analytical framework that accounts for uncertainty

This study relies on exploratory spatial data analysis (ESDA) within a broader framework to investigate the uncertainty of reported risk locations. The framework includes descriptive statistics, statistical testing, statistical modeling, and geographic information systems (GIS) as detailed in Fig. 1. ESDA extends the exploratory data analysis of Tukey [26] to focus on both aspatial and spatial effects relating to the accuracy with which locations were reported. ESDA has been previously applied to analyze the spatial patterns of locations and a variety of different health-related outcomes [27–34].

The statistical analysis component indicated in Fig. 1 utilizes descriptive statistics, chi-squared testing, multinomial logistic modeling, region fixed effects, and sandwich clustered errors. Descriptive statistics were employed to outline and summarize survey respondent characteristics. Statistical testing utilized chi-squared tests to identify significant variables relating to the accuracy of reported locations. Multinomial logistic regression models were then used to relate the reported accuracy of locations. The GIS component in Fig. 1 reflects spatial data management and facilitates ESDA capabilities to link spatial patterns and the accuracy of location reporting. GIS was further employed to delineate cluster region boundaries based on fixed effects. Finally, GIS supported the visualization of statistical analysis and significance. Building upon these key components, the uncertainty analytics component in Fig. 1 indicates methods developed and applied to account for survey-based locational imprecision. These uncertainty analytics were employed to generate spatial confidence regions, delineating the uncertainty associated with key predictors to better drive targeted interventions in the future. Finally, the spatial analytics component in Fig. 1 includes approaches to identify clusters of activity, taking into account location uncertainty. Technical details now follow.

### Data management and processing

Locations were joined with demographic characteristics by a unique identifier (PID). Then, activities that were carried forward from a previously marked location were updated based on the reported accuracy level. This was performed using GIS to identify locations reported by the same respondent at the same location. Reporting accuracy was then classified as “Exact”, “Not Exact”, and “Not Comfortable Marking Location”. Demographic and activity characteristics were aggregated in order to identify general trends in the relationship between geographic, location-based, and respondent attributes on reporting accuracy. The demographic characteristics selected for the study as potential predictors of reporting accuracy were race, education level, annual income, HIV status, having come out to someone, partnership status, being born in the US, and age. These variables were selected based on the conceptual framework, suggesting that location reporting accuracy may be shaped by social position, access to resources, comfort with disclosure, life-course factors, and the stigma or sensitivity attached to particular places and behaviors. Race/ethnicity was included because prior work has suggested that mobility, neighborhood exposure, institutional trust, and vulnerability to surveillance may differ across groups [35–37]. Education and income were used as indicators of socioeconomic position that may map literacy, technology use, residential stability, and comfort with survey participation. Age was included as recall, familiarity with digital interfaces and routine activity patterns may vary over the life course. Partnership status and having come out to someone were included because social support, privacy concerns, and disclosure-related experiences may influence willingness to report sensitive locations. HIV status was included because HIV-related stigma and engagement with care may shape comfort with reporting prevention and risk-related locations. US-born status was used as nativity may capture differences in neighborhood familiarity, migration histories, and experiences interacting with institutions. Location type was included because the stigma, regularity, and memorability of activities were hypothesized to differ across different activity locations. Finally, regions were included to account for broader geographic context, including neighborhood composition, service environments, and place-based stigma that may influence location reporting accuracy.

Manual imputation and predictive mean matching were used to fill respondent non-response when necessary, based on the other information provided. Several demographic variables contained small numbers of missing or non-response values; 4 respondents refused to report their income levels, 1 respondent refused to report their education level, 1 respondent refused to report both their education level and their outness, 2 respondents refused to report their outness, and 1 respondent refused to report if they had been born in the US. A structured imputation strategy was therefore implemented using auxiliary demographic and behavioral variables available in the dataset to impute the missing levels among the 6 individuals. For income and education variables, rule-based manual imputation was applied when sufficient supporting information was available. For example, respondents with missing income values were assigned to the “*less than $50,000*” category when other indicators suggested lower socioeconomic status, such as reporting homelessness, being unemployed, or enrollment in public health insurance programs, or reliance on public assistance. Similarly, respondents who failed to provide an education level were recorded as “*Less than college*” when other socioeconomic indicators suggested lower educational attainment. For the outness measure, which was obtained by directly asking respondents if they had ever come out to anyone, missing responses (NA and refuse to answer) were evaluated using multiple behavioral indicators related to sexual identity disclosure and relationship status. Finally, for the small number of cases with missing or refused responses for nativity, predictive mean matching was implemented using respondent-level similarity to impute nativity from the most similar respondent.

Activity locations (referred to as location type) were reported for a respondent’s current home, their home 12 months ago, their home 5 years ago, their primary medical care, their most recent HIV test, their most recent STD test, and their pharmacy. Respondents further reported up to the three most recent places where they had engaged in sex with their three most recent sexual partners, up to their three most recent places where they engaged in substance use, and their primary and secondary social locations. To account for multiple activity locations reported for different survey respondents, organizations, by their general activity type, were linked to multiple reported locations. The individual activity type characteristic (named type) was still maintained, which maintained the identification of the most recent, second most recent, third most recent, primary, secondary, and home 5 months and home 12 months ago for each location. GIS was used to identify the region within which each location was reported, and subsequently used as a fixed effect in the regression models.

### Analysis

Descriptive statistics were derived to describe the demographic characteristics of study respondents and the types of locations collected in the survey. Chi-squared testing was used to compare observed results with expected results to determine if there is a difference between the observed identified predictors of accuracy and the expected location reporting accuracy to determine if predictors appeared to be statistically significantly associated with the reported accuracy of locations (Table 1).

**Table 1:** Study variable definitions and descriptions

**Variable**	**Description**	**Type**
General Reporting Accuracy	Generalized measure of location reporting accuracy indicated by survey respondent*Levels:* Exact, Not Exact, Not Comfortable the Exact Location (abbreviated as Not Comfortable and Not Comfortable in regression tables)	Categorical
Race/Ethnicity	Aggregated respondent ethnicity*Levels:* Non-Hispanic Black, Hispanic, Other	Categorical
Education	Aggregated highest level of respondent-reported education*Levels:* Less than college, some college (or technical school), college degree or more (completed bachelor, master, or associate degree, etc)	Categorical
Annual Income	Aggregated respondent annual income level*Levels:* Less than $50,000, $50,000 or more	Categorical
Partnership	Respondent partnership status based on if the respondent is currently in a relationship with a same sex partner*Levels:* Living Together or Married, Boyfriend (not married and answer yes to *Is/was your recent sexual partner someone that you feel or felt committed to above all others (someone you might call a boyfriend, partner, life partner, significant other, husband)?*), Not Boyfriend (participants who have recently engaged in sexual activity but do not consider their recent sexual partner to be a committed relationship), No Recent Sexual Activity (participants who have not recently engaged in sexual activity and do not have a partner)	Categorical
US Born Self	Refers to respondent having been born in the US or not.*Levels:* Yes, No	Categorical
Has Come Out	Refers to if a respondent has come out to someone, meaning that they have told someone else they are gay, bisexual or sexually attracted to men.*Levels:* Yes, No	Categorical
Age	Respondent age	Integer
HIV Status	Refers to respondent HIV status based on mSTUDY HIV testing*Levels:* Living with HIV (HIV+), HIV negative (HIV-), Missing	Categorical
General Location Type	Refers to the activity occurring at individual locations*Levels:* Sex, drug, social, home, medical care	Categorical
Multiple Location Identifier	Identifies individual locations reported for each general type (respondents may report more than one location per activity)*For drug and sex locations:* Three most recent locations*For home locations:* Home 5 years ago, home 12 months ago, and current home location*For social locations:* Primary and secondary locations*For medical care locations:* Most recent HIV test location, most recent STD test location, regular medical care location, and pharmacy location	Categorical
Region	Fixed effect based on the region within which the reported location is contained with.*Levels:* Region Name	Categorical

Multinomial logistic regression was employed to evaluate the likelihood of a location being reported exactly relative to other characteristics. Three general models were considered:

1 $$\begin{aligned}log(\frac{P(Y=c)}{P(Y=Exact)})&=\upbeta +\upbeta Race/Ethnicity +\upbeta Education+\upbeta Annual\,Income\\&+\upbeta Age+\upbeta Partnership+\upbeta Has\,Come\,Out\\&+\upbeta US\,Born+\upbeta HIV\,Status +\upvarepsilon\end{aligned}$$

2 $$\begin{aligned}log(\frac{P(Y=c)}{P(Y=Exact)})&=\upbeta +\upbeta Race/Ethnicity +\upbeta Education+\upbeta Annual\,Income\\&+\upbeta Age+\upbeta Partnership+\upbeta Has\,Come\,Out+\upbeta US\,Born\\&+\upbeta HIV\,Status +\upbeta Location\,Type+\varepsilon\end{aligned}$$

3 $$\begin{aligned}log(\frac{P(Y=c)}{P(Y=Exact)})&=\upbeta +\upbeta Race/Ethnicity +\upbeta Education+\upbeta Annual\,Income\\&+\upbeta Age+\upbeta Partnership+\upbeta Has\,Come\,Out+\upbeta US\,Born+\upbeta HIV\,Status \\&+\upbeta Location\,Type+\upbeta Region+\varepsilon\end{aligned}$$

where *P(Y* = *c)* is the probability of being in the non-reference category* c* (Not exact or Not comfortable marking the exact location) and β are the coefficients for the predictor variables given the outcome category.

All models included demographic characteristics as predictors, with variations in the subsequent models. Model (1) included only demographic characteristics. Model (2) added the location type term, and relates to the general activity type of the reported location, hypothesizing that the type of activity occurring at a location may influence reporting accuracy. Model (3) adds region fixed effects, hypothesizing that the geographic context of a location within a larger region may influence the reporting accuracy. The categorical dependent variable with multiple levels, in this case, the accuracy of reported locations, made multinomial logistic well suited for comparison across accuracy levels to determine if different demographics are more likely to intentionally not report exact locations.

As respondents could report a varying number of locations for each given general activity type, standard errors in all models were clustered by respondent PID to account for the multiple observations by individuals. Using a cluster-robust covariance matrix, or cluster VCE, the independent and identically distributed assumption of independent errors is relaxed and replaced with the assumption of independence between errors, thus allowing for errors to be correlated within clusters [38, 39]. The cluster errors are a generalization of the ‘sandwich’ method used to compute heteroskedasticity-robust standard errors in all three models [38], and can be specified as follows:

4 $$Var(\varepsilon_{c})=VCE(\hat{\beta})=(X^{\prime}WX)^{-1}(\sum\nolimits_{g=1}^{G}{X^{\prime}}_{g}{u}_{g}u{^{\prime}}_{g}{X}_{g}(X^{\prime}WX)^{-1}$$

where *X* is the matrix of model predictors, *W* is the weight matrix, ${\hat{u}}_{g}$ are the residuals for cluster *g*, ${X}_{g}$ represents the predictors for observations with cluster *g* and the summation aggregates across clusters.

To account for multiple hypothesis testing across the large number of coefficient estimates produced by the multinomial models, *p*-values were adjusted using the Benjamini–Hochberg false discovery rate (FDR) procedure. Multinomial logistic regression produces a separate coefficient estimate for each predictor across each non-reference outcome, resulting in a substantial number of simultaneous statistical tests. Without adjustment, conducting many tests increases the likelihood of identifying statistically significant associations simply due to random variation. The Benjamini–Hochberg procedure controls the expected proportion of false positives among the set of rejected hypotheses rather than strictly controlling the probability of any false positives, as in more conservative procedures such as Bonferroni correction [40]. The FDR adjustment was applied across all coefficient-level tests from the multinomial models, excluding the intercept terms, and adjusted *p*-values were then used to determine which demographic characteristics, activity-type predictors, and regional fixed effects remained significantly associated after accounting for the multiplicity of statistical tests. Because the activity-type variable represents a multi-level categorical factor, evaluating the significance of individual coefficients alone does not fully capture whether activity type as a whole contributes to explaining variation in reporting accuracy. To address this, an omnibus likelihood-ratio (LR) test was conducted to address the joint contribution of the activity type predictor. The LR test compares the fit of a restricted model that excludes the activity type term and a full model that includes it. This nested model comparison evaluates whether the set of activity type coefficients improves model fit. The LR test also provides a complementary inferential framework to the coefficient-level tests by determining whether activity type is associated with overall reporting accuracy, even if individual activity contrasts do not remain statistically significant after multiple testing correction.

The models were then compared to determine if the inclusion of the location type term affects the predictor estimates of the demographic characteristics. This was performed to better understand the impact of stigmatized activities on the generalized location reporting accuracy and to determine the influence of stigmatized activities on individuals. The multinomial modeling was performed first using all location types (1) and (2) to better understand what general characteristics influence general reporting accuracy overall, after which the model was refitted to assess the influence of geographic location (3). Model performance and relative fit were evaluated using both in-sample and out-of-sample comparison metrics. For in-sample comparisons, Akaike Information Criterion (AIC), Bayesian Information Criterion (BIC), and model log-likelihood values were computed for each model specification, allowing comparison of model goodness-of-fit while accounting for model complexity, thereby enabling assessment of whether the addition of location type and regional fixed effects meaningfully improves model performance. To further evaluate model stability, grouped k-fold cross-validation was conducted. Because multiple locations could be reported by the same individual, cross-validation folds were constructed by grouping observations by respondent. This ensured that all observations from a given respondent were placed within the same training or test fold, thereby preventing information leakage across folds and providing a more realistic assessment of model generalizability. Five-fold grouped cross-validation was used, and models were repeatedly trained on four folds of data and evaluated on the remaining fold. Prediction performance was summarized using log-loss, which measures the accuracy of predicted probabilities for the multinomial outcomes, and classification accuracy defined as the proportion of correctly predicted outcome categories. Mean accuracy across folds was then used to compare model performance. Model results were then compared and mapped to show neighborhoods significantly related to reduced likelihood and neighborhoods significantly related to the likelihood of reporting the location as either not exact or not being comfortable reporting the exact location.

## Results

Among 206 respondents in the analysis sample, 97 respondents or 47.1% reported all locations with the same accuracy, while 109 respondents or 52.9% reported locations with varying levels of accuracy. Specifically, a total of 77 respondents reported all locations with an exact accuracy level, 19 respondents reported all locations with not exact accuracy, and 1 respondent reported feeling not comfortable to mark the exact location for all locations they reported. On average, respondents reported 10.3 locations across the 5 different location types surveyed. Of these reported locations, respondents reported locations with exact accuracy 6.9 times, not exact locations being reported an average of 2.9 times, and reporting not feeling comfortable marking the exact location being reported an average of 0.4 times out of the average number of total locations reported by each respondent. Of the 2112 reported locations included in the analytical sample, 69.8% of activity locations were indicated as exact, while 26.1% were not exact, with 4.0% of locations having a general reported accuracy of not comfortable marking location.

Table 2 outlines the demographic characteristics of the 206 respondents included in the study sample. A majority (55.3%) identified as Hispanic, while 33.5% were Non-Hispanic Black, and 11.6% identified as other races/ethnicities. Nearly half (49.5%) of the respondents had post-secondary education, while 27.2% had a higher degree and 23.3% had less than a post-secondary education. Most respondents (80.1%) reported an annual income of less than $50,000, and over half (57.8%) were living with HIV. The majority of respondents (85%) were US-born, and less than a third (30.1%) reported being in a relationship, either living with, being married to, or considering their most recent sexual partner to be their boyfriend. The average age of respondents was 37 years, with a standard deviation of 7 years.

**Table 2:** Distribution of demographic characteristics by respondent

**Variable / Category**	**Count**	**Percentage**
Race/Ethnicity		
Non-Hispanic Black	69	33.5
Hispanic	114	55.3
Other	23	11.16
Education		
Less than College	48	23.3
Some College	102	49.5
College Degree or More	56	27.2
Annual Income		
Less than $50,000	165	80.1
$50,000 or more	41	19.9
HIV Status		
Living with HIV (HIV+)	119	57.8
HIV Negative (HIV-)	80	38.8
Missing	7	3.4
US Born Self		
Yes	175	85.0
No	31	15.0
Has Come Out		
Yes	188	91.3
No	18	8.7
Partnership		
Living Together or Married	14	6.8
Boyfriend	48	23.3
Not Boyfriend	94	45.6
No Recent Sexual Partner	50	24.3
Age	Average	Standard Deviation
Age	37.0	7.0

Table 3 illustrates the distribution of the reported locations by the activity occurring at the location. The five primary categories surveyed were medical care, home, drug-related, sex-related, and social locations. The table reveals notable differences in the frequency and distribution of reported locations within these categories, reflecting both the study design and survey respondent behaviors. As the survey asked participants to report up to four different locations for medical care activities, medical care locations accounted for the highest number of reports. Home locations followed closely with 561 reported locations. Drug-related locations accounted for 410 reported locations. Among medical care, home, and drug-related locations, these categories had fairly consistent numbers across the counts of the individual locations reported within, with drug-related locations having a near-equal distribution across the three most recent drug-reported locations. Sex related locations (325 reported locations) and social locations (187 reported locations) showed much greater variability in the distribution of reported locations within the general categories. Among both sex related locations, fewer respondents reported secondary sex related locations, with even fewer reporting tertiary sex related locations. Among social locations, a noticeable drop was found as only about 62% of respondents who reported their primary social locations also reported their secondary social locations.

**Table 3:** Distribution of reported locations by activity type

**Location Type**	**Number**
Medical Care	629
Primary medical care	189
Pharmacy	159
Recent HIV test	143
Recent STD test	138
Home	561
Current home	193
Home 12 months ago	189
Home 5 years ago	179
Drug	410
Most recent drug location	139
Second most recent drug location	137
Third most recent drug location	134
Sex	325
Most recent sex location	138
Second most recent sex location	99
Third most recent sex location	88
Social	187
Primary Social Location	115
Secondary Social Location	72
Total Locations Reported	2112

**Fig. 2:**
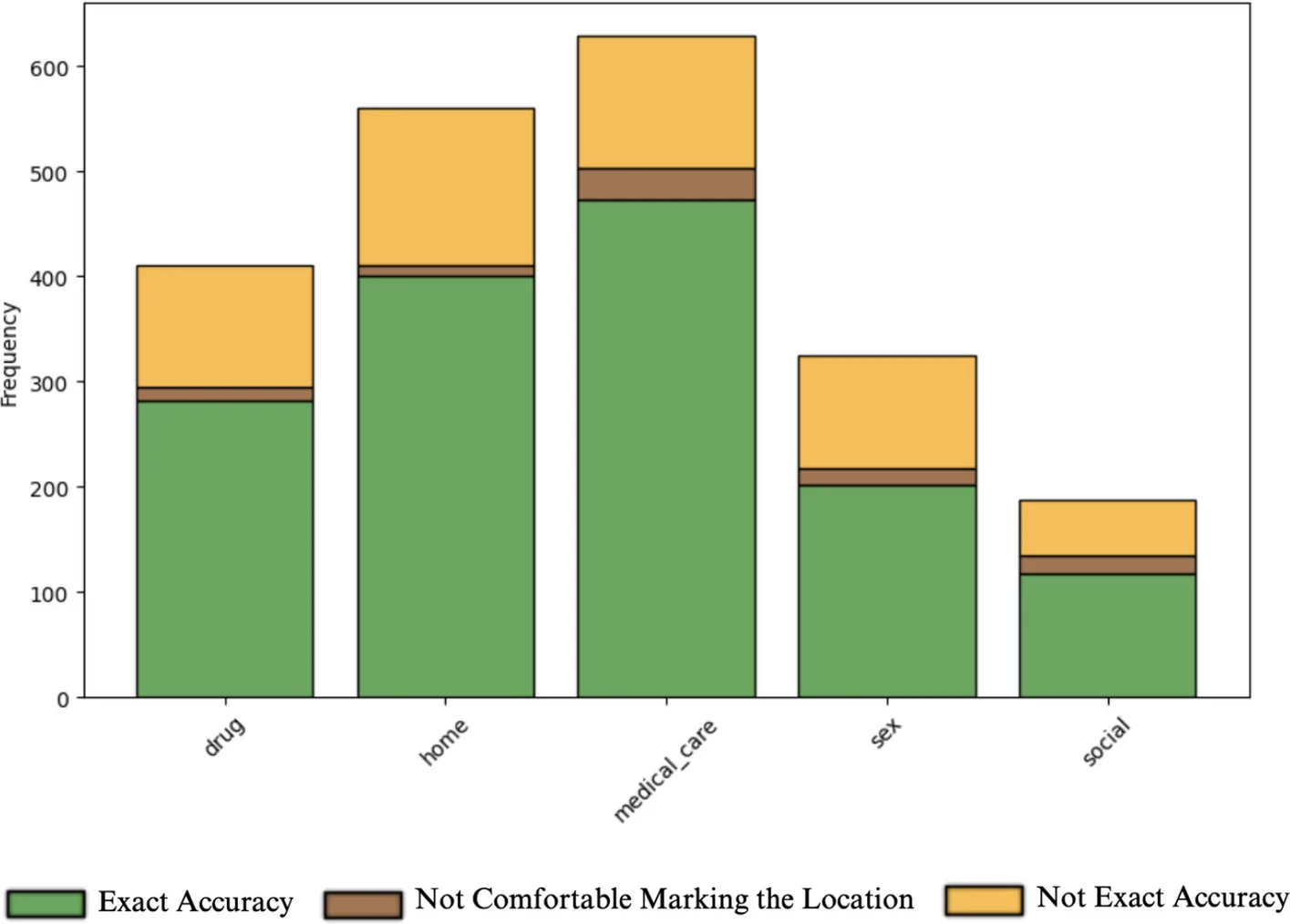
Accuracy of location reporting, for all locations, by general type

As shown below in Fig. 2, location reporting accuracy varied by the general type of the reported locations. Medical care locations had the highest frequency of locations reported exactly, while social locations had the lowest. Although general location reporting accuracy appears fairly constant among locations reported with exact accuracy, more variation is seen across the different location types among those reported as not exact. The largest variations by type were seen in locations where respondents indicated they did not feel comfortable marking the exact location. When considering the proportion of locations reported, medical care locations had the greatest proportion of locations being reported as exact (0.75), while sex locations had the greatest proportion of not being reported as not exact (0.33), and social locations had the greatest proportion of not being comfortable marking the exact location (0.085). Social locations also had the smallest proportion of being reported exactly (0.53), and home locations had the smallest proportion of not feeling comfortable marking the exact location (0.019), while medical care locations also had the smallest proportion of not exact accuracy (0.20).

As shown below in Fig. 2, location reporting accuracy varied by the general type of the reported locations. Medical care locations had the highest frequency of locations reported exactly, while social locations had the lowest. Although general location reporting accuracy appears fairly constant among locations reported with exact accuracy, more variation is seen across the different location types among those reported as not exact. The largest variations by type were seen in locations where respondents indicated they did not feel comfortable marking the exact location. When considering the proportion of locations reported, medical care locations had the greatest proportion of locations being reported as exact (0.75), while sex locations had the greatest proportion of not being reported as not exact (0.33), and social locations had the greatest proportion of not being comfortable marking the exact location (0.085). Social locations also had the smallest proportion of being reported exactly (0.53), and home locations had the smallest proportion of not feeling comfortable marking the exact location (0.019), while medical care locations also had the smallest proportion of not exact accuracy (0.20).

**Fig. 3:**
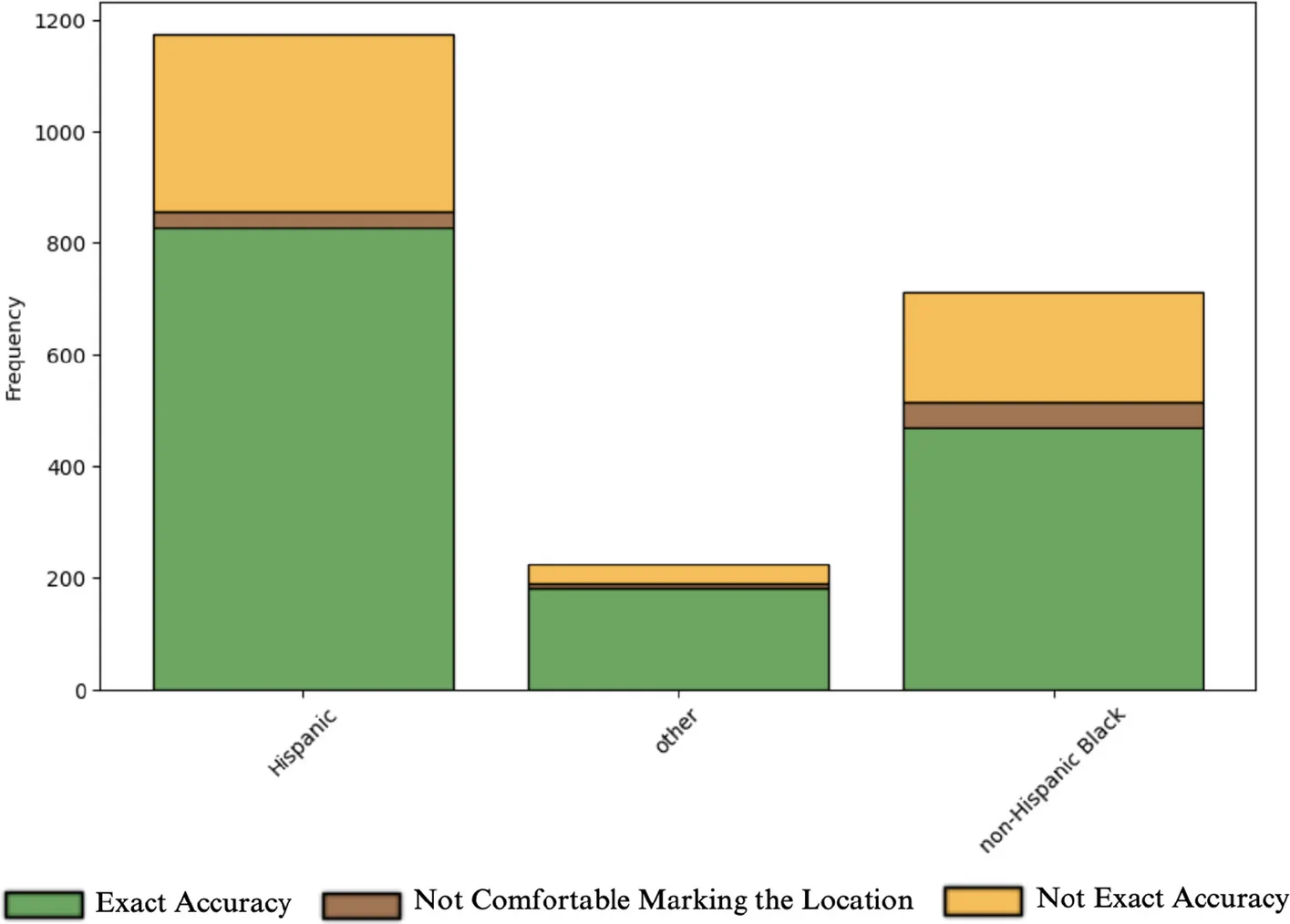
Accuracy of location reporting, for all locations, by ethnicity

As shown below in Fig. 3, general location reporting accuracy also varied by the ethnicity of the respondent. Hispanic respondents reported the highest frequency of locations and reported the highest frequency of locations with exact accuracy. However, respondents who were neither Black nor Hispanic reported the greatest proportion of reported locations as exact, while also reporting the smallest proportion of locations as not exact. Non-Hispanic Black respondents reported the greatest proportion of not feeling comfortable marking exact locations, while Hispanic respondents reported the smallest proportion. Non-Hispanic non-Black respondents reported the greatest proportion of locations as exact (0.80), while non-Hispanic Black individuals reported the greatest proportions of not exact locations (0.28) and not being comfortable marking the exact location (0.06). Non-Hispanic Black respondents reported the lowest proportions of exact locations (0.65), while Hispanic respondents reported the smallest proportion of not feeling comfortable marking the exact location (0.03). Non-Hispanic non-Black respondents also reported the smallest proportion of not exact locations (0.15).

The relationship between activity type, respondent characteristics, and general location reporting accuracy was evaluated (chi-squared test). This assessed whether the accuracy of reported locations was independent of various demographic variables, general activity types, and the ability to report multiple locations per respondent for each general activity. The results are summarized in Table 4. The findings indicate that, at a significance level of 0.05, the null hypothesis—that the tested variables are not related to the reporting accuracy—could not be rejected for respondent income and the ability to report multiple locations. This provides sufficient evidence to conclude that accuracy does not have a significant relationship with the respondent’s annual income level. This also suggests that whether a location was the most frequently or recently visited for a given activity type does not influence the reporting accuracy of the location. For the remaining variables, the null hypothesis was rejected, indicating significant relationships among accuracy and respondent race/ethnicity, education, partnership, age, being born in the US, having come out to someone, and general activity of the reported location. 

**Table 4:** Chi-squared test for associations with general reporting accuracy

**Variable**	***P*** **-Value**	X^2^
Race/Ethnicity	0.00	29.95
Education	0.00	96.10
Annual Income	0.57	1.12
Partnership	0.00	109.58
Age	0.00	391.62
US Born	0.00	26.66
Has Come Out	0.00	24.44
Age	0.00	227.35
HIV Status	0.00	16.54
General Location Type	0.00	53.07
Multiple Location Identifier	0.99	10.06

Prior to model estimation, diagnostic checks were conducted to evaluate data quality and the assumptions of the multinomial logistic framework. The analytic sample included all 2,112 observations, as no cases were removed due to missing covariate data. The outcome distribution was moderately imbalanced (69.8% exact, 26.1% not exact, and 4.0% not comfortable marking the exact location), and the relatively small size of the not comfortable category was considered when interpreting the results. The clustering structure was also examined, revealing 206 respondents with a median of 11 observations per respondent. Geographic group sizes were uneven, with several spare regional categories that may yield less stable estimates in models including regional fixed effects. Multicollinearity diagnostics indicated low variance inflation factors across all predictors (maximum VIF = 3.610), suggesting that multicollinearity was not a concern. All multinomial models converged with no missing coefficients or standard errors and no extreme parameter estimates indicative of separation or instability. Finally, predicted probabilities were evaluated and found to indicate numerically stable model estimation. In sum, these diagnostics suggest that the multinomial models were statistically well behaved, although the outcome imbalance and sparse geographic categories remain important considerations for interpretation.

Model (1), which analyzed reporting accuracy with only demographic characteristics, revealed key demographic predictors of location reporting accuracy. Non-Hispanic Black respondents were found to be significantly more likely to report discomfort in marking exact locations compared to Hispanic respondents. Conversely, respondents with post-secondary education and those who lived with or were married to their partners were significantly less likely to report discomfort in reporting exact locations compared to respondents with college degrees or those who considered their most recent sexual partner to be their boyfriend. Additionally, individuals living with or being married to their partner and those whose HIV status was missing were less likely to report exact locations than respondents with boyfriends or HIV-positive respondents. However, after applying the Benjamini–Hochberg FDR correction across all coefficient tests, only a limited subset of these associations remained statistically significant. In particular, respondents with some college education and respondents who were married to or living with their partner remained significantly less likely to report discomfort in marking an exact location. In contrast, the previously observed association for non-Hispanic Black respondents was no longer statistically significant after the FDR adjustment. Similarly, none of the demographic predictors remained statistically significant once the multiple-testing correction was applied.

When the general activity type of the reported location was added to Model (2), it was evident that the activity occurring at a location influences reporting accuracy. Social locations were associated with greater discomfort in reporting exact locations, while medical care locations were associated with respondents being less likely to feel discomfort when reporting the location, relative to drug-related locations. The predictors previously identified in Model (1) remained significant, though slight shifts in estimates and errors were observed, indicating nuanced effects when activity type was included in the model. When performed on the entire location reporting dataset, Model (1) had an AIC of 2982.47 and a log likelihood of −1463.23, while Model (2) had an AIC of 2954.11 and a log likelihood of −1441.05. Both the AIC and log-likelihood ratio suggest that the addition of location type in Model (2) provided a significantly better fit than Model (1). Likelihood-ratio tests further confirmed that the inclusion of activity type significantly improved model fit (LR χ2 = 44.4, df = 8, *p* < 0.001), demonstrating that reporting accuracy varies systematically by activity type. Post-hoc comparisons suggested that social locations were associated with greater discomfort in reporting exact locations compared to substance use locations, although this comparison did not remain statistically significant after false discovery rate correction. Other activity types showed weaker and non-significant differences relative to substance use locations. However, after applying the FDR correction, none of the activity-type predictors remained statistically significant. Although social locations were associated with higher odds of reporting discomfort, and medical care locations were associated with lower odds of reporting locations as not exact compared to substance use locations, these associations did not remain statistically significant after controlling for multiple testing; only social locations remained marginally significantly associated with the likelihood of not being comfortable reporting exact locations. However, the significant demographic predictors remained statistically significant after the FDR correction was applied.

When geographic location was included in the model using the formulation specified in Model (3), no changes to previously identified significant predictors were observed for locations where respondents were not comfortable marking the exact location. However, the addition of the region fixed effects found that being married or living with a partner was no longer significantly associated with a lower likelihood of reporting not exact locations. Additionally, sex locations were found to be associated with respondents being significantly more likely to report not exact locations following the inclusion of geographic location. All other significant predictors previously identified in Model (2) remained significant; however, their coefficients were modified by the inclusion of the regions. Model (3) had an AIC of 2896.67 and a log likelihood of −1378.33, suggesting that the inclusion of region fixed effects significantly improved fit. After applying the FDR correction, the main statistically significant predictors remained concentrated in association with not being comfortable marking the exact location. Respondents with some college education and those who were married to or living with their partner continued to exhibit significantly lower odds of reporting discomfort in marking an exact location. In contrast, the previously observed associations between location type and location reporting accuracy were no longer statistically significant. Several region fixed effects remained significantly associated with the odds of not feeling comfortable reporting an exact location, indicating that reporting accuracy varied geographically across regions. Although Model (3) improved in-sample model fit relative to Models (1) and (2), grouped-cross-validation results suggested that the added complexity of the region fixed effects did not improve out-of-sample predictive performance and may have reduced model generalizability. Although Model (3) produced the best in-sample fit based on AIC and log-likelihood, grouped cross-validation suggested that the added complexity of region fixed effects reduced predictive performance, with mean accuracy declining to 0.650 compared to 0.695 for Model (1) and 0.689 for Model (2), suggesting that while regional fixed effects improve in-sample model fit, they may not generalize well to new observations (Table 5).

**Table 5:**
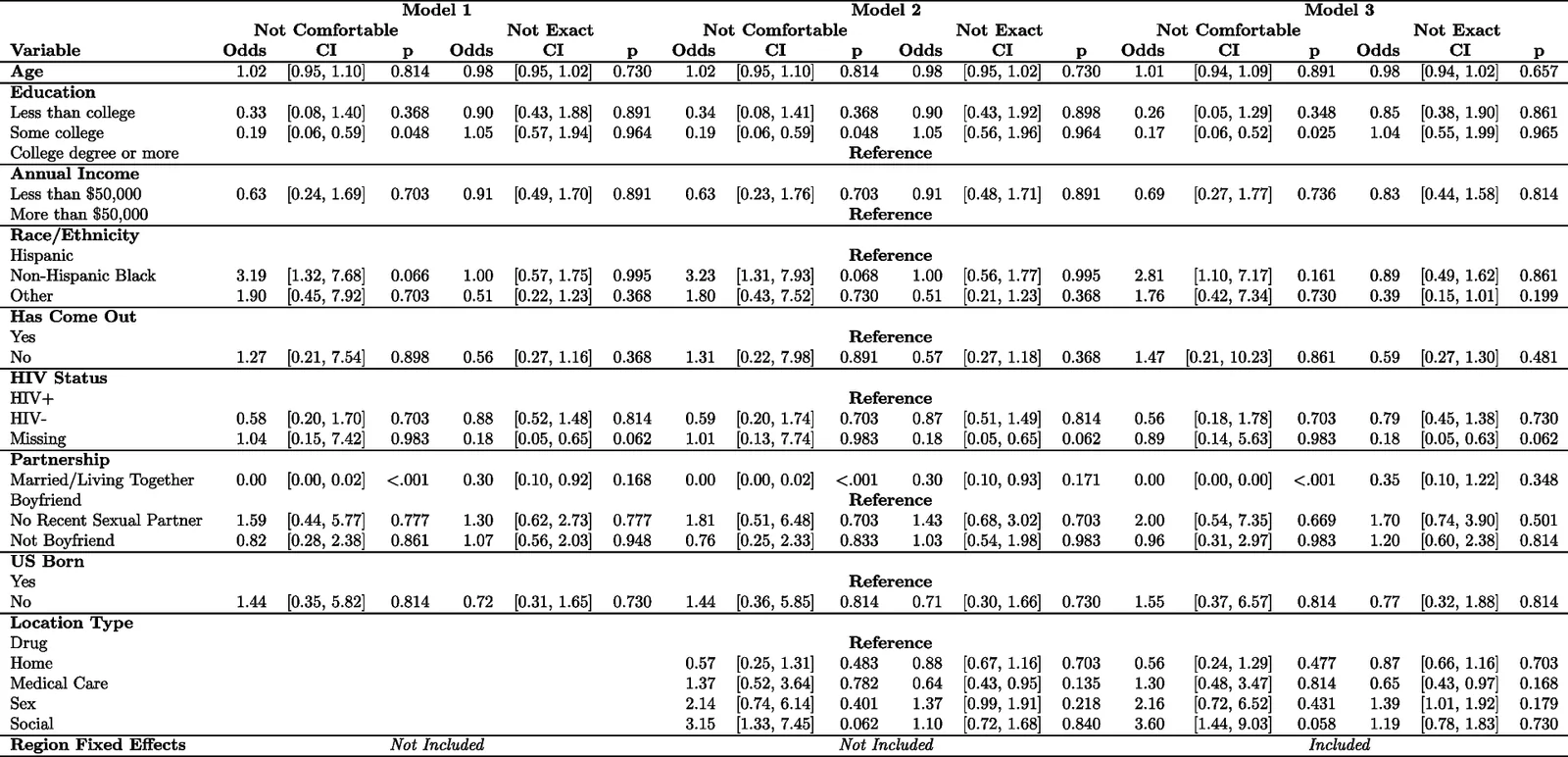
Results of multinomial models of location reporting accuracy (FDR-adjusted *p*-values)

Reported *p*-values are Benjamini–Hochberg false discovery rate (FDR) adjusted *p*-values

**Fig. 4:**
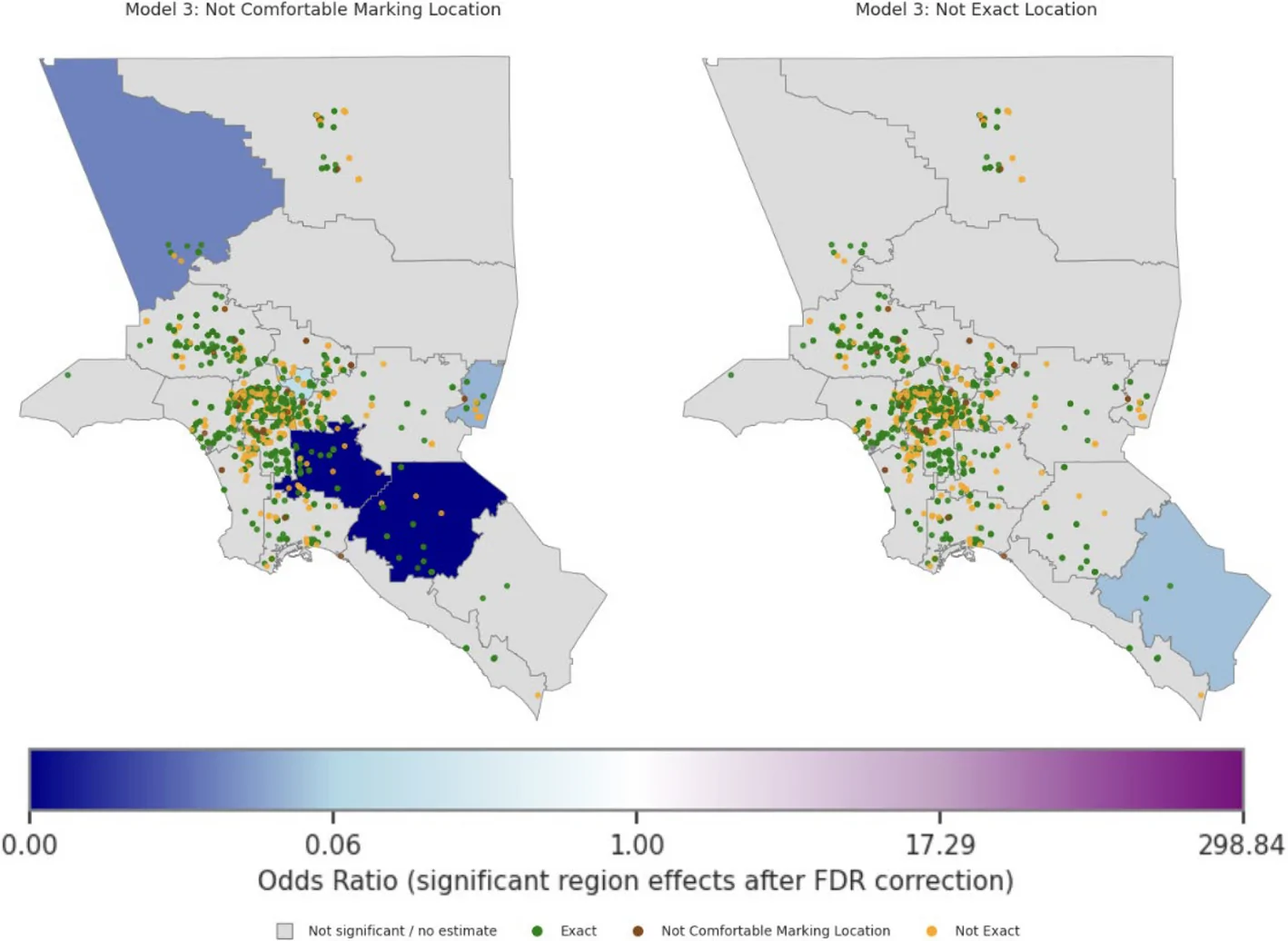
Significant region fixed effects from accuracy model (3)

The inclusion of fixed effects revealed that five regions were significantly associated with discomfort in marking exact locations, while only one region was significantly associated with not marking exact locations. Notably, none of the regions were significantly associated with both outcomes after applying the FDR correction, indicating that geographic factors associated with discomfort in reporting exact locations differ from those associated with imprecise location reporting. This suggests that regional variation plays a nuanced role in location reporting accuracy. Figure 4 illustrates the geographic distribution of these significant regions, and the consistency across location type, but also some variation in regional effects. The strength and direction of these effects differed by location type and accuracy measure, highlighting the complexity of geographic influences on reporting behavior. While previous models found that demographic and location activity type factors may drive individual-level differences in the accuracy of respondent-reported locations, regional fixed effects were found to help capture broader geographic trends, improve model fit, and reveal more nuanced patterns in reporting behavior across Los Angeles County. Specifically, the regional fixed effects suggest that some parts of Los Angeles County are associated with systematically lower levels of discomfort when reporting exact locations, while a smaller number of regions appear to influence the likelihood that locations are reported imprecisely.

## Discussion

This study demonstrates that the accuracy of reported locations is shaped by a combination of individual demographic characteristics, the type of activity occurring at a given location, and the geographic context describing the regional position of the location. Model (1), focusing only on demographic predictors, revealed that non-Hispanic Black respondents were more likely to report discomfort in marking exact locations compared to Hispanic respondents. Conversely, those with post-secondary education, married or cohabitating, or individuals with a missing HIV status were less likely to express discomfort. These trends emerged when all locations were modeled simultaneously, underscoring the influence of demographic factors on reporting behavior. When activity type was included in Model (2), distinct patterns emerged. Social locations were associated with respondents being more likely to report discomfort in reporting the exact location, while medical locations were more likely to be reported with exact accuracy compared to drug-related locations. Likelihood-ratio tests comparing models with and without the activity type indicated that inclusion of activity type significantly improved model fit overall, suggesting that reporting accuracy varies systematically by activity type. However, despite the strong overall effect, none of the individual activity type relationships remained statistically significant after applying false discovery rate correction, suggesting that while activity type as a whole influences reporting accuracy, no singular category independently explains this variation once multiple testing is considered. Results of multinomial models of location reporting accuracy (FDR-adjusted *p*-values)

Several possible mechanisms underlie these findings, although they should be interpreted cautiously. The greater likelihood that Non-Hispanic Black respondents reported discomfort in marking exact locations may reflect broader structural and historical contexts rather than individual reluctance alone. For example, unequal exposure to policing, surveillance, criminalization of certain spaces or behaviors, and long-standing mistrust of institutions may contribute to greater caution when identifying sensitive locations. These dynamics may be especially relevant when reporting locations tied to stigmatized activities; likewise, neighborhood context may matter as respondents may perceive some places as more socially exposed, which could reduce willingness to mark an exact location. The observed associations for education, partnership status, and activity type may also reflect differences in resources, privacy concerns, and the social meaning of places. Respondents with more education may be more comfortable navigating the map interface or more familiar with formal addresses and neighborhood boundaries, which could improve reporting precision. Partnership status may capture differences in household stability, social support, or concerns about disclosing sensitive activities. Activity type likely influences reporting accuracy through multiple pathways, including the regularity of visits, the stigma attached to the activity, the distinctiveness of the place, and the respondent’s comfort in disclosing it.

The chi-square test on the term indicating how recently a specific location had been visited by the respondent revealed no significant association with the reporting accuracy, which suggests that recency alone was not strongly associated with reporting accuracy in the sample and indicates that no clear relationship between recency and reported accuracy was observed. However, the significance of the general activity type term in both the chi-squared test and modeling suggests that activity type occurring at a location plays a role in reporting, although the associations between respondent characteristics and reporting accuracy may differ across activity contexts. Overall, this study found that reporting accuracy is driven by complex associations among demographic, activity-related, and geographic factors. This study found that reporting accuracy varies significantly based on respondent characteristics such as race, education, partnership status, and HIV status. Moreover, this variation is influenced by the type of activity being reported. For example, individuals with higher education may report substance use locations less accurately if they are reluctant to associate these activities with their home environments. Although some activity-type contrasts suggested patterns consistent with stigma or contextual sensitivity, these individual contrasts did not remain statistically significant after controlling for multiple testing, thereby suggesting that activity context matters broadly, but its influence is distributed across categories rather than within any singular activity type. Incorporating regional fixed effects not only improved model performance but also provided insights into how geographic context is associated with the relationship between accuracy and demographic characteristics. Importantly, spatial uncertainty was primarily linked to these demographic and place-based factors.

The inclusion of region fixed effects in Model (3) highlighted the role of geographic context. Incorporating regional fixed effects revealed that geographic context significantly affects reporting accuracy. This finding underscores that accuracy is influenced by who a person is, what they are doing, and where they are doing it. Notably, adding regional fixed effects to models adjusted the significance of demographic predictors related to the likelihood of not marking the exact location, but did not adjust the significance of predictors related to the likelihood of not feeling comfortable marking the exact location. Rather than demonstrating mediation, these results indicate that region location was independently associated with reporting accuracy after adjustment for demographic and activity-related characteristics, and that inclusion of region fixed effects changes the magnitude and significance of other coefficients. This suggests that geographic context has a differential effect on accuracy, relating more to the precision of location reporting than to the comfort of location reporting. While not affecting the significance of predictors of the likelihood of not feeling comfortable marking the exact location, the addition of region fixed effects reduced the magnitude of the effect of race/ethnicity. This aligns with prior research (e.g., [41]) showing that spatial clustering of activity spaces varies by race and socioeconomic status, influenced by residential segregation. The inclusion of region fixed effects also showed that when considering geographic location, some location types not previously identified as significant were indeed significant. Nevertheless, once FDR corrections were applied, most activity-type associations did not remain statistically significant, reinforcing the interpretation that the primary contribution of activity type lies in its overall influence on reporting accuracy rather than in the effects of specific activity categories. This may suggest that the role of geographic context varies by the level of stigma or regularity of the activity occurring, more research is needed to support this.

Despite the attenuation of statistical significance following the Benjamini–Hochberg false discovery rate correction, the overall pattern of results remained largely consistent with the unadjusted models when considering a more permissive threshold of ɑ = 0.1. At this level, many of the predictors identified as significant prior to correction continued to meet this criterion, suggesting that the underlying associations are not spurious but may instead be sensitive to multiple testing adjustments. The Benjamini–Hochberg procedure operates by ranking *p*-values and comparing each to an increasing threshold based on its rank, thereby controlling the expected proportion of false discoveries among rejected hypotheses rather than the probability of any false positive. As a result, the procedure is inherently sensitive to both the number of simultaneous tests conducted and the distribution of *p*-values assessing those tests. In settings such as this study, where multinomial models generate a large number of coefficient-level tests and outcome categories are unevenly distributed, statistical power is reduced, and *p*-values are less likely to fall below the increasingly stringent FDR correction threshold. Consequently, the attenuation of significance reflects the joint influence of multiple testing burden, outcome imbalance, and sample size constraints rather than the absence of meaningful underlying relationships.

As individuals report locations differently based on their individual level demographic characteristics, the activity occurring at the location, and the geographic location, analyses that rely only on these reported locations without accounting for these differences risk introducing systematic bias. For instance, non-Hispanic Black respondents were more likely to express discomfort in location reporting, so policies driven by analyses not considering the differential likelihoods in reporting exact locations may fail to adequately capture the activity spaces of non-Hispanic Black individuals, thereby failing to accurately reduce disparities in HIV incidence and prevalence. Similarly, differences in the accuracy reporting for certain types of locations (e.g., sex locations vs. medical care locations) can skew findings, creating artificial patterns or masking true relationships. If these variations are not explicitly controlled for or considered in modeling, the resulting analysis may misinterpret spatial or behavioral trends, perpetuate inaccuracies, and lead to flawed conclusions or ineffective interventions. The finding that activity type is significant as a factor but not through individual contrasts further highlights the need for models that consider broader contextual influences rather than focusing solely on activity-specific categories. Developing methods to account for such reporting differences is essential to minimize bias and ensure robust, reliable results. If some groups or types of locations are less likely to be reported precisely, hotspot analyses based on self-reported locations may under-identify areas where prevention and care resources are most needed or may misclassify the spatial distribution of risk. Improving the validity of location reporting, therefore, has practical implications for the equitable targeting of HIV prevention and care resources, and possible approaches include strengthening rapport and trust during data collection, clearly communicating confidentiality protections, allowing respondents to report approximate locations using graduated accuracy levels, incorporating follow-up prompts that distinguish privacy concerns from recall difficulties, and partnering with community organizations to improve engagement.

However, the findings are limited by the study’s sample and context. Data were collected from a cohort of sexual minority men in Los Angeles County, rather than from a probabilistic sample of the broader population of SMM. Thus, the findings should not be interpreted as representative of all SMM in Los Angeles or elsewhere. Additionally, although the sample included respondents across racial/ethnic groups, the race/ethnicity variable was analytically grouped into Hispanic, Non-Hispanic Black, and Other, which necessarily compresses heterogeneity within the latter category. Data was collected during a period that overlapped with the COVID-19 pandemic, a period that significantly altered activity spaces. Thus, reported locations may not fully represent current behaviors, and reporting accuracy may have been influenced by pandemic-related factors such as income disparities or changes in mobility patterns. Future research aims to expand the dataset to include a more diverse and representative sample. Additionally, more robust models should be developed to incorporate a broader range of individual characteristics for each location type. These models highlight the need for methodology to facilitate the creation of spatial confidence intervals to delineate the likely true location of reported activities. By simulating hotspot analyses within these confidence regions, researchers can quantify the impact of spatial uncertainty on identifying HIV risk hotspots.

Several additional limitations should also be noted. First, important contextual influences such as experience stigma, perceived surveillance, institutional mistrust, neighborhood safety, and prior interactions with policing were not directly measured and therefore could not be included as predictors even though they are likely to influence reporting accuracy. Second, as the data is self-reported, the results may be affected by social desirability bias and other forms of response bias, which is especially relevant in HIV research where respondents may under-report stigmatized behaviors or locations, over-report socially desirable behaviors, or selectively reduce the precision of sensitive information. Third, this study is geographically specific to Los Angeles County, where service availability, urban form, transportation systems, and neighborhood stigma may differ substantially from those in other regions. Finally, the sample was relatively socioeconomically concentrated, which may limit variability in some predictors and reduce generalizability to populations with broader socioeconomic diversity. These limitations suggest that the observed associations should be interpreted as context-specific and future work should reevaluate these associations in other populations and settings.

## Conclusions

Geographic research often assumes that spatial uncertainty is minimal and evenly distributed, but this study demonstrates that it is shaped by demographic, activity-based, and geographic factors. The findings further suggest that spatial uncertainty in self-reported HIV-related activity locations is systematic, not random, and instead reflects broader social and geographic patterns. Therefore, spatial uncertainty has important implications for the identification of HIV risk hotspots and the design of place-based interventions. Addressing disparities in HIV incidence and prevalence rates, particularly through place-based interventions, must acknowledge these differences. Given the variation in spatial uncertainty across demographic groups and activity contexts, analyses relying on self-reported locations may systematically misidentify or underrepresent areas where prevention and care resources are most needed. As a result, interventions informed by these analyses may fail to adequately reach populations experiencing the greatest HIV-related vulnerabilities, thereby contributing to the persistence of disparities in HIV incidence and prevention access. Moreover, the presence of regional variation in reporting accuracy further indicates that spatial uncertainty is not randomly distributed across space, but instead reflects underlying geographic patterns in reporting behavior. This study emphasizes the need for new methods that account for the uneven distribution of spatial uncertainty in survey-reported locations. Although this study identified systematic variation in spatial uncertainty, the effects of this on analytical conclusions have not yet been explored. Future work will examine how spatial uncertainty influences hotspot identification and spatial epidemiologic analyses. Future research should also focus on incorporating spatial uncertainty directly into hotspot identification and spatial epidemiological modeling to improve the validity, equity, and effectiveness of place-based prevention studies. Without such approaches, demographic differences in reporting accuracy will continue to bias analyses, potentially compromising the effectiveness of interventions designed to address disparities in HIV incidence and prevalence.

## Data Availability

All data related to the participants, including information collected during the cognitive interviews, are strictly confidential. Access to these data is restricted to the University of California, Los Angeles and mSTUDY team to ensure the privacy and confidentiality of the participants. A copy of the survey can be made available upon request to the lead author. Additional information about participants in the cognitive interviews, such as demographic data, is not available based on the approved institutional review board guidelines.
